# Qualitative Assessment of the Feasibility, Usability, and Acceptability of a Mobile Client Data App for Community-Based Maternal, Neonatal, and Child Care in Rural Ghana

**DOI:** 10.1155/2016/2515420

**Published:** 2016-12-14

**Authors:** Jessica D. Rothstein, Larissa Jennings, Anitha Moorthy, Fan Yang, Lisa Gee, Karen Romano, David Hutchful, Alain B. Labrique, Amnesty E. LeFevre

**Affiliations:** ^1^Department of International Health, Johns Hopkins Bloomberg School of Public Health, 615 N. Wolfe Street, Room E5030, Baltimore, MD 21205, USA; ^2^Johns Hopkins University Global mHealth Initiative, 615 N. Wolfe Street, Room 5635, Baltimore, MD 21205, USA; ^3^Grameen Foundation, No. 25 Labone Crescent, Accra, Ghana

## Abstract

Mobile phone applications may enhance the delivery of critical health services and the accuracy of health service data. Yet, the opinions and experiences of frontline health workers on using mobile apps to track pregnant and recently delivered women are underreported. This evaluation qualitatively assessed the feasibility, usability, and acceptability of a mobile Client Data App for maternal, neonatal, and child client data management by community health nurses (CHNs) in rural Ghana. The mobile app enabled CHNs to enter, summarize, and query client data. It also sent visit reminders for clients and provided a mechanism to report level of care to district officers. Fourteen interviews and two focus groups with CHNs, midwives, and district health officers were conducted, coded, and thematically analyzed. Results indicated that the app was easily integrated into care, improved CHN productivity, and was acceptable due to its capacity to facilitate client follow-up, data reporting, and decision-making. However, the feasibility and usability of the app were hindered by high client volumes, staff shortages, and software and device challenges. Successful integration of mobile client data apps for frontline health workers in rural and resource-poor settings requires real-time monitoring, program investments, and targeted changes in human resources.

## 1. Introduction

An estimated 66% of maternal deaths and 50% of deaths in children under the age of 5 occurred in sub-Saharan Africa in 2015 [1, 2]. Although Ghana's health indicators largely surpass many African nations, the country has faced significant challenges in improving maternal and child survival. Despite nearly halving the number of maternal deaths since 1990, approximately 319 women die each year out of 100,000 live births from pregnancy- and birth-related complications—a figure nearly twice that of the Millennium Development Goal [1, 3]. Among children under five, while overall deaths have declined by 51% since 1990, neonatal deaths occurring within 28 days after birth have largely remained stagnant, changing only from 30 to 29 deaths per 1,000 live births [2, 4].

A vast majority of maternal, neonatal, and child deaths are preventable [1, 5–8]. However, limited access and utilization of skilled antenatal, delivery, and postpartum care services hinder the timely and safe delivery of life-saving interventions [6–9]. In addition, reliable and comprehensive data on the implementation of maternal, neonatal, and child health services are needed to monitor and ensure that life-saving services are provided across the care continuum [10–12]. However, the inability of traditional health information systems to track new mothers and babies once they leave health facilities as well as inadequate communication between health workers at different levels of the health system has led to slower progress towards maternal, neonatal, and child health goals [13–15].

Global penetration of mobile phones has the potential to optimize delivery of high quality maternal, neonatal, and child health information and access to services [16–21]. In particular, mobile apps, or software applications developed specifically for use on small, wireless computing devices such as smartphones or tablets, have been shown to improve the quality of care provided to pregnant and recently delivered women through electronic decision support [17, 18, 22], health worker planning [23, 24], and data collection and reporting [19, 25–27]. The reported ease of use and adaptability of mobile apps (or applications) for diverse populations also makes them an attractive, low-cost platform for developing health education and clinical care strategies [28–30]. Nonetheless, to increase efficacy and sustainability of mobile health applications, previous studies have underscored the importance of ensuring high user acceptance and usability of the technological innovation [31–33]. User-centered design and robust needs assessments are recognized as best practices for development technology [34]. Equipping frontline health workers with mobile phone applications may enhance the delivery of maternal, neonatal, and child health services as well as the accuracy of data capture and recording [16, 26, 35, 36]. Yet, little research exists on health worker perceptions of mobile applications intended to improve maternal, neonatal, and child data management, despite user views being critical to product uptake and implementation [37–39]. In addition, the opinions and experiences of frontline health workers in sub-Saharan Africa on using mobile applications to track pregnant women and mother-infant pairs are underreported. Such information is critical for improving the long-term impact and sustainability of mobile health (mHealth) strategies for clinical and community health settings.

*Evaluation Objective*. This evaluation aimed to qualitatively examine the feasibility, usability, and acceptability of the Client Data Application (or Client Data App) that was part of the Mobile Technology for Community Health (MOTECH) program to support frontline health workers in the delivery of maternal, neonatal, and child care in rural Ghana.

**Figure 1:**
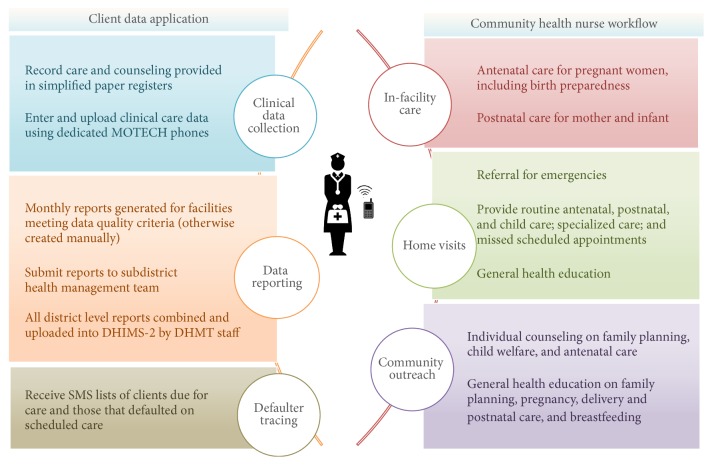
Functional summary of MOTECH's mobile Client Data App.

The MOTECH program was initiated in 2009 as a partnership between the Ghana Health Service, Grameen Foundation, and Columbia University Mailman School of Public Health to leverage mobile technology to increase quantity and quality of maternal and infant care in rural areas and ultimately improve health outcomes. The Client Data App enabled community health nurses (CHNs) to use mobile phones to digitize care provided to women and children and thereby track pregnant women and mother-infant pairs needing care (Figure 1). CHNs then received reminders and alerts about clients' upcoming and missed care. Parallel care reminders and alerts, in addition to other actionable health information, were sent to pregnant women and mothers with infants less than 12 months of age as part of a mutually supportive “Mobile Midwife” program within MOTECH [40]. A separate evaluation, not discussed in this manuscript, is planned to examine health workers' views on MOTECH's Mobile Midwife service.

## 2. Methods

### 2.1. Setting

Data were collected in 2014 in two districts: Awutu Senya and Gomoa West. MOTECH was implemented in Awutu Senya in 2011 and then replicated in Gomoa West and three other districts in 2013. Both districts are located in Ghana's Central Region and have among the highest rates of under-five and neonatal mortality in the country [4, 40]. This region additionally has significant human resource shortages. In the Central Region of Ghana, there are over 25,000 individuals to one doctor compared to the nationwide ratio of nearly 12,000 individuals to one doctor [41, 42]. The region also suffers from high vacancy rates of nurse-midwives trained to manage basic and emergency obstetric care [43–45]. Under these circumstances, mobile technologies that expand the reach of health workers are critical to overcoming barriers to care. Although telecommunication connectivity in the Central Region can be unreliable and mobile phones are not ubiquitous, phone ownership and access are high among health workers [46].

Maternal, neonatal, and child health services within Ghana's health system are primarily delivered via public health centers and community health posts. The health centers are staffed primarily by skilled health professionals (such as nurses and midwives) who offer comprehensive preventive and basic curative services, including minor surgeries and uncomplicated deliveries. In contrast, as part of Ghana's Community-based Health Planning and Services (CHPS) Initiative, community health posts are staffed by lower-skilled CHNs who provide health education, outreach and counseling, and basic curative services to clients via home visits and facility-based care [47]. Community health posts (referred to locally as CHPS facilities) are typically staffed by 2 to 3 CHNs who are required to have completed a two-year postsecondary certificate program in obstetrics as well as general and community health nursing. MOTECH Ghana was originally designed for use in community health posts and later extended to the health centers, hospitals, and other private health facilities. Within the two evaluation districts, MOTECH was implemented in a total of 46 facilities, including 35 community health posts and 11 health centers.

### 2.2. Description of the Client Data App

**Figure 2:**
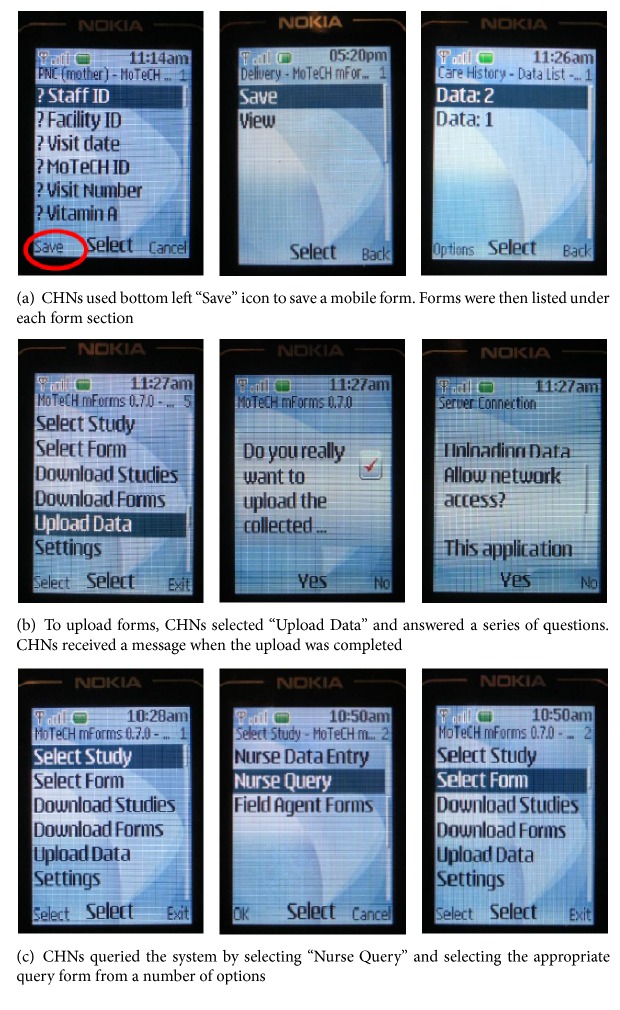
Screen shots of the MOTECH Client Data App's saving, uploading, and query forms.

The mobile Client Data App used in this evaluation was delivered by low-cost GSM mobile Nokia 1680 and Nokia Asha 200 feature phones provided by Ghana Health Service, which helped CHNs and other users to digitize and track care delivered to mother-infant pairs in their area. The system's architecture was based on field-tested open-source software, including OpenXData for mobile data collection and OpenMRS for electronic medical records [48]. The client data system used a Java 2 Platform Micro Edition (J2ME) application to capture and store client data. All clients were assigned a unique MOTECH identification (ID) number upon registration to protect confidentiality and enable tracking across multiple facilities. During client encounters, CHNs first recorded care provided using five “simplified paper registers,” which were developed by MOTECH to condense more than a dozen registers and streamline data collection. CHNs later entered data into digital forms on their mobile phones (Figure 2(a)). General packet radio service (GPRS) data channels were used to transfer these data from the phone to a central clinical data system that was stored on the MOTECH server (Figure 2(b)).

The data app system then crosschecked uploaded clinical information on timing and type of care given with national guidelines to estimate specific due dates for routine care. As a result, health workers received a weekly list via short message service (SMS) of pregnant clients and mother-infant pairs in their catchment area who were either due for or defaulted on care. CHNs were also able to query client data, enabling them to retrieve lists of defaulters or women due to deliver in the upcoming week, and to search for details about individual clients (Figure 2(c)). In addition, the Client Data App generated preselected monthly health reports that were required for national reporting, if client data were at least 85% complete and accurate three consecutive months. Previously, monthly health reports were numerous, redundant, and compiled by hand. Therefore, the Client Data App was intended to improve accuracy and processing speed.

The MOTECH developers designed the Client Data App for low-skilled health providers in rural and resource-poor settings. To account for anticipated power and mobile network breaks, the Java-enabled Nokia handsets allowed for mobile forms to be completed and stored offline for uploading at a later time. Phones had dual subscriber identity module (SIM) capacity and were equipped with SIM cards from two different mobile operators in case of network or congestion problems [46]. Field testing during the prototyping stage served to align the app's features with user needs, including the simplification of data entry using check boxes, radio buttons, lists, and number fields. CHNs received in-person training as well as a detailed training manual. They could also refer to a MOTECH call center for technical assistance. Monthly prepaid airtime units were provided to all users to upload information. The Client Data App's interface was available for use in English. Phones were password-protected, and user authentication schemes were built into Java forms to maintain confidentiality of client data [46].

### 2.3. Measurement

Qualitative in-depth interviews and focus groups were used to examine health worker perceptions on the Client Data App's feasibility, usability, and acceptability. For purposes of this evaluation, we defined the assessment areas as follows:* Feasibility* was defined as whether implementation of the Client Data App was easily and conveniently done, accounting for advantages and disadvantages to integrating the application into routine workflow.* Usability* was defined as whether the Client Data App could be used by CHNs to adequately record, track, and summarize data, including whether it functioned in a way that enhanced productivity or led to unproductive tasks due to errors.* Acceptability* was defined as whether CHNs and other stakeholders found the Client Data App likeable, including its interface and navigation features. These definitions were derived from similar prior research that qualitatively assessed user experiences for mHealth applications [28, 49–55].

### 2.4. Data Collection

Data were collected at three levels of the health system: community health posts (referred to locally as CHPS facilities), health centers, and district health directorates. Semistructured interview guides were used at all levels. In-depth interviews with CHNs and midwives asked them to describe perceived benefits and drawbacks of the Client Data App, as well as their experience using it during clinic and community outreach activities. CHNs and midwives were also asked to assess advantages and disadvantages of using the Client Data App for tasks such as recording care, tracking clients, and verifying data with automatic health reports. Skilled nurses working at the health centers did not use the Client Data App and therefore were not recruited for data collection. Interviews with district health directors and district health information officers explored how the Client Data App affected the quality of data provided by CHNs and their ability to use and supervise submission of monthly health reports. Questions also examined views regarding local and scaled-up implementation. Other district health directorate staff, including disease control officers and public health officers, who did not engage with the Client Data App were not interviewed. Focus groups with CHNs were included to further investigate findings from the individual interviews and to obtain CHNs' recommendations for modifying the MOTECH data management system. Focus groups with midwives are not conducted given the limited number of midwives available at participating sites.

All interviews and focus groups were conducted in English by a local Ghanaian and a US researcher, both with experience conducting qualitative research. The Ghanaian researcher occasionally translated local terminology in Akan used by participants. The interviews were conducted at community health posts and health centers and ranged from 40 to 120 minutes. Focus groups were conducted in a centrally located and neutral space. The discussions and lasted approximately 90 minutes. Each interview and focus group was digitally recorded and transcribed verbatim.

### 2.5. Sample Selection

One health center and three community health posts were randomly selected from each of the two participating districts. Purposive sampling was then used to identify CHNs, midwives, district health information officers, and district health directors with a minimum of six months experience using MOTECH's Client Data App. The target interview sample size was 14 individuals, representing one CHN and one midwife per health center, one CHN each from three community health posts in each district, and the district health director and district health information officer in each district. The target focus group sample size was two groups each with 7 to 8 CHNs. Given resources available, this sample size was expected to enable the evaluation to reach saturation in which no new findings emerged [56].

### 2.6. Analysis

A qualitative thematic analysis was conducted by two public health graduate students. Interview and focus group transcripts were manually coded using* a priori *topical codes according to the evaluation's three assessment areas: feasibility, usability, and acceptability. Emergent subcodes were then developed based on patterns within each concept and which were relevant to the literature. We then followed an iterative process of developing a codebook, identifying salient themes, and integrating core findings [57]. When new themes were identified throughout this process, transcripts were reanalyzed to find evidence that verified or modified those themes. Later-stage interviews and focus groups were used to validate responses among member participants [58]. We also confirmed findings based on feedback from MOTECH implementation partners during various stages of data collection and analysis [59].

### 2.7. Ethics Approval

The evaluation was approved by the institutional review board at the Johns Hopkins Bloomberg School of Public Health in Baltimore, Maryland, USA. As part of the MOTECH implementation plan, the Ghana Health Service in Accra, Ghana, also approved the evaluation's activities. All participants provided oral informed consent prior to data collection. This manuscript complies with the mHealth evidence reporting and assessment (mERA) checklist on reporting of health interventions using mobile technologies [60].

## 3. Results

**Figure 3:**
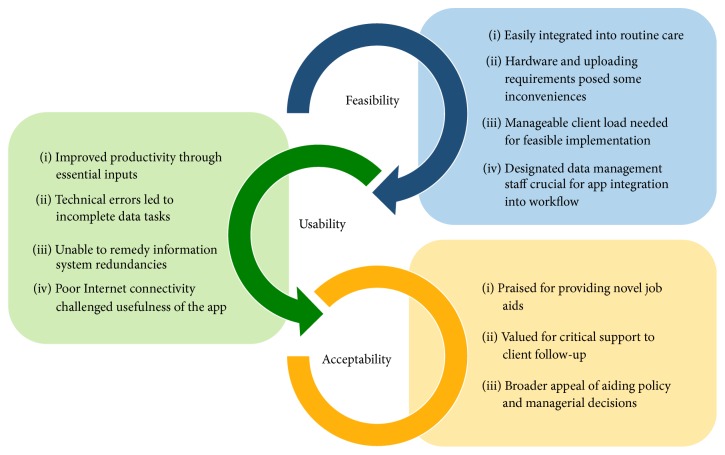
Summary of key qualitative findings by assessment area.

Several findings emerged from our analyses, revealing both the achievements and challenges of MOTECH's implementation of the Client Data App. The emergent themes within each of the three assessment areas (feasibility, usability, and acceptability) are illustrated below with exemplary quotations. Key findings are summarized in Figure 3.

### 3.1. Participant Characteristics

A total of 14 individual interviews were conducted, representing eight CHNs and two midwives as first-line users of the Client Data App, as well as two district health information officers and two district health directors (Table 1). Two focus groups were additionally carried out, consisting of a total of 15 CHNs. The majority of participants were female and within the ages of 26 to 30 years.

### 3.2. Feasibility

We defined feasibility as whether implementation of the Client Data App was easily and conveniently achieved, accounting for advantages and disadvantages to integrating the application into routine care.

#### 3.2.1. Easily Integrated into Routine Care

Participants generally reported that the Client Data App was easily integrated into their workflow and data capture. CHNs were able to incorporate registration of eligible individuals, including issuing their MOTECH IDs, into their client visits. CHNs also noted being able to readily incorporate information provided by MOTECH alerts into their weekly routines. Based on the ease of integration, users were able to envision the Client Data App becoming a permanent part of their work.

*When a pregnant woman comes here, you start reviewing her records. When there is a new client, you give her an ANC booklet; you fill the form for her, taking the vitals of the client. Then you take their mobile phone number and register the woman into MOTECH …. (CHN, health center)*

*… Every Monday morning,… the MOTECH system sends us alert messages to remind us on those who are due for care. And those who are defaulters too … So, every Monday morning I checked the phone and gets tracked on the data, and the details on those who are due for care and those who are defaulters and follow them and give the care to them. (CHN, community health post)*

*We just hope that the MOTECH project comes to stay forever and incorporate it into Ghana Health Service, forever. (District health information officer)*

#### 3.2.2. Hardware and Uploading Requirements Posed Some Inconveniences

However, limited mobile network connectivity in more rural areas of the district posed significant challenges to uploading client data. Consequently, CHNs had to preoccupy themselves with locating places with adequate connectivity in order to upload the mobile forms. This presented an added inconvenience, particularly for those CHNs working in remote areas. For some CHNs, accommodating network connectivity challenges also required them to extend their workday.

*We also have problems with network connectivity. For example, so the uploading may be a challenge. And sometimes they're typed, but it doesn't go through. Sometimes it doesn't go through at all. You have to go and climb a tree. (District health information officer)*

*We try to find a place where the network is good. For example, when the network is good, you can upload everything. Let's say, my house, the network is good, I have to take the phone and upload there. (CHN, health center)*

CHNs and other users additionally expressed concerns about the hardware used, as the small size and tedious typing functionality of the Nokia 1680 phones made data entry time-consuming. This rendered the Client Data App as a less feasible option in the long run, especially at high-volume health centers. Requests to be equipped with larger screen devices with keypads such as laptops or tablets were given by over half of interview participants, who stressed the challenges associated with their current handsets.

*The numbers are quite huge numbers. If you are doing about 30 a day … They have to enter them. Thirty people. It's tedious. So maybe they get a laptop or something bigger …. (District health information officer)*

*They also should give us laptop, or tablet, as we are typing, we can't speed it. I would prefer a smart phone because in smart phone, when you type something you don't have type everything, it will just [automatically] fill. (CHN, health center)*

Another challenge involved the omitted generation of automated reports for some facilities because reporting completeness and accuracy had not reached 85% for the required three consecutive months. Limitations in meeting completion and accuracy thresholds and the corresponding failure to receive automated reports left many users frustrated.

*Tomorrow I will be thinking I have to submit the report by Monday. Then I will be sitting here and do[ing] the uploads. If we are supposed to submit on Monday, there is a lot to do. But the reports are not coming, oh. We did three months and so automation … will generate reports for you. I was so happy, but up to now I didn't see anything. (CHN, community health post)*

#### 3.2.3. Manageable Client Load Needed for Feasible Implementation

Feasible implementation was also hindered by a shortage of CHNs or other data entry staff, especially in health centers where client volume outweighed staff availability. High client caseloads interfered with CHNs' capacity to submit clinical data and receive timely alerts on clients who were due for or missed care. The significant clinical responsibilities of trained midwives at health centers and the unpredictability of their workdays also posed challenges to the consistent use of the Client Data App. The imbalance between client loads and health worker availability was compounded by high CHN attrition rates, which required retraining of staff on the Client Data App.

*Because we are the health center, unlike the CHPS zone, concerning our volume, after the outreach, you have to do all the uploads. We cannot do the uploads rapidly because of our workload. Maybe they can take out the health centers or have something done to help because our workload is intense. (CHN, health center)*

*… By the end of the day, maybe you have a lady who haven't deliver[ed] yet, so after you close the antenatal phase, you have to go to the labor ward and be monitoring this labor case. Then by the end of the day,… you are coming back to load the MOTECH … In fact it's a headache. (Midwife, health center)*

*… In non-urban settings, like this place, staff attrition is very high. … For the past about three years, we've been receiving an average of about 20 community health nurses every year. … These are new people. So they don't know anything about MOTECH. They have to be trained. (District health director)*

#### 3.2.4. Designated Data Management Staff Crucial for App Integration into Workflow

Many participants commented that the feasibility of the Client Data App would be improved if staff were designated to data management rather than responsible for multiple care tasks, especially during community outreach activities. Others suggested that health worker shortages could be overcome by training health extension workers (who had previously been discontinued) for routine data entry. In addition, some participants proposed recruiting other MOTECH staff to assist with routine data entry during care provision.

*I do have some problems with … recording the care given. Sometimes …, at the child welfare clinic, I have to take the height and the MUAC [mid-upper arm circumference] of the babies. … I will be doing the weighing, the immunizing and the recording, and a whole lot of it. Sometimes I even forgot to take the MUAC or the height … There is no help. We need some nurses to help us. (CHN, community health post)*

*If we can get other cadre of staff, who are not necessarily well trained, so that they can do this ….We did train some health extension workers, and then other staff … [but]—they are not professionals. We did train them, but unfortunately they've all been exited out of the system, so that has compounded the problem. (District health director)*

*Would that be possible? To train personnel from MOTECH, and then they join us on the field? Or join us in our health facility? Because basically in Ghana, our work is very broad. Very huge. So, together with MOTECH help, it should end. (District health information officer)*

### 3.3. Usability

We defined usability as whether the Client Data App could be used by CHNs to adequately record, track, and sum data, including whether it functioned in a way that enhanced productivity or led to unproductive tasks due to errors.

#### 3.3.1. Improved Productivity through Essential Inputs

CHNs reported that MOTECH's simplified paper registers were a significant improvement over the previous data collection process. The mobile forms were perceived as straightforward and easily navigable. CHNs reported that they were generally able to transfer clinical information from the simplified registers to the mobile forms in approximately two minutes. In addition, by enabling CHNs to avoid redundant paper registers, the Client Data App saved time and allowed for more case entries per day and week.

*I think MOTECH has made our work much easier. Before there is a lot of writing, then after MOTECH, maybe the important information will be portioned, so maybe when you write, it's easier. (CHN, health center)*

*We don't rule lines anymore and the immunizations, we can write the actual number, everything else is there. (CHN, health center)*

#### 3.3.2. Technical Errors Led to Incomplete Data Tasks

Nevertheless, the Client Data App's usability was hampered by technical errors which interfered with completing registration and data submission. The most commonly reported error involved identical MOTECH ID numbers designated to different clients. User delays resulting from these errors were also exacerbated by difficulties reaching the technical support team. In addition, data entry was often interrupted when the software froze unexpectedly.

*They will say “the MOTECH ID is already in use.” The MOTECH ID says it's new. So I stopped registering them. I have five clients who are not registered now. If I use those MOTECH ID numbers, they will just send me errors. (CHN, community health post)*

*Sometimes there are errors. I uploaded, and I called the call center, but they don't respond. Like I have been contacting them but no answer. (CHN, community health post)*

*It's sometimes ….an experience … on the phone. I think sometimes it freeze, when you are working on some forms, you realize that it stops … then you realize it's freeze. (CHN, health center)*

#### 3.3.3. Unable to Remedy All of the Information System Redundancies

Usability challenges also arose because MOTECH was not interoperable with the national health information system. While the integration of MOTECH data with the District Health Information Management System 2 (DHIMS-2) was beyond the program's resources and scope, participants expressed frustration with this disconnect. The process of recording and summarizing client data was also delayed because it was not possible for MOTECH to automate all of the monthly reports for qualifying facilities.

*Mind you, we have been advocating that if the MOTECH system and the DHIMS-2 would be on the same platform - so that this one will fit into this. So you don't have to print out, before you re-enter. That will save a lot of time. (District health information officer)*

*We do the entry at the sub-district level, all the facilities in MOTECH. Change our phone and get us [a] tablet. Then we can do the DHIMS-2 ourselves at the sub-district level. (CHN, community health post)*

*There is nothing about antenatal care. Even though they take [mobile] data on antenatal care, which they upload. There's nothing on family planning—they also take [mobile] data on family planning. So, the core areas which we think should be there because of MOTECH for the [mobile] reports are omitted. (District health information officer)*

Finally, some of the information needed to identify and track clients in their communities was not readily available through the Client Data App's alerts or queries. CHNs suggested including client addresses and travel instructions to ease follow-up in remote areas and the overall usability of the application.

*The reminder is good, but the difficulty in tracing the defaulters is the address. To get the address, we still have to go back to the simplified registers to locate the defaulters. When the phone is in your pocket and you look at it, you see the person … [but] it only comes with the name and the care. The addresses are not there. (CHN, health center)*

#### 3.3.4. Poor Internet Connectivity Challenged the Usefulness of the App

The usefulness of the Client Data App was likewise affected by interruptions in network connectivity. Inconsistent connectivity led to difficulties in uploading clinical data and/or errors. This delayed receipt of timely alerts of clients requiring care. The discrepancy between data collection and submission also hindered the Client Data App's tracking functionality and the accuracy of the aggregate numbers in the automated monthly reports.

*The internet connection is not that stable … It is time-consuming when you are doing the uploads because you have to try it, try it, try it. When the network is good, we just do it once and it hits the system … The network is not stable. The thing indicates it's connecting, for five minutes—it's still connecting. So you have to wait, waiting, waiting, waiting  ⋯ (CHN, community health post)*

*Because sometimes we upload the forms but it doesn't go through. … It means it wouldn't be captured by the system. The report generated from the form will be different from what we have here. It is a challenge we have. … Yesterday the phone I uploaded, it didn't go through. It is only today …. (CHN, community health post)*

In order to minimize delays due to connectivity interruptions, one CHN suggested that MOTECH incorporate additional feedback loops between users and the central database.

*… Maybe on Mondays, if they can send us what we've uploaded for us to cross check. If it's not uploaded we will re-send it … It will be easier and faster than just waiting for them. Otherwise we always complain about the automated generated report, because it's not tallying what's really happened. So given the reminders, they should also give us the reminders of the uploads. (CHN, health center)*

### 3.4. Acceptability

Acceptability was defined as whether CHNs and other stakeholders found the Client Data App likeable, including its interface and navigation features.

#### 3.4.1. Praised for Providing Novel Job Aids

The Client Data App was acceptable to users given that it introduced new tools to assist health workers in performing their assigned tasks. In general, CHNs were satisfied with MOTECH's alert system. In particular, CHNs and those at community health posts found that the automated monthly reports eased their workload for data reporting and motivated them to capture and use data.

*Sometimes, when you go to the outreach … it's difficult for you to go to the register and find out which community you went to and which not. When you go to the reminders on MOTECH, it will just tell you because you uploaded the information after the outreach. So MOTECH has been really helpful to us, especially in the remote areas. (CHN, community health post)*

*It is so rewarding when at the end of month, you receive your computer-generated reports. And then you don't have to go through the registers and do the targets. Even that, there's some incentive for those who receive the computer degrees and reports …. (District health information officer)*

Conversely, CHNs who worked at facilities that were not receiving the automated reports due to incomplete submitted data were less enthusiastic about the Client Data App. They expressed a desire to see these new job aids materialize.

*I wish MOTECH system could generate all the reports for me. Then I will be motivated to upload everyday! I'd be more motivated if I see the reports. (CHN, community health post)*

#### 3.4.2. Valued for Critical Support to Client Follow-Up

The Client Data App was also valued for improving the efficiency of client follow-up through weekly reminders, which eliminated the need for CHNs to conduct time-consuming searches through paper registers. In addition, CHNs noted that the weekly defaulter lists helped structure their community outreach and home visit schedules, maximizing the follow-up care provided.

*Before MOTECH, sometimes we don't trace defaulters. It's better to trace. It's very helpful. Because if you don't trace, you don't know what's happening to your clients. Everything about the clients is on the phone. The vaccine [and] the routine. (CHN, community health post)*

*Before the MOTECH, we don't have the reminders, I write it somewhere else, sometimes I remember it. Sometimes you remember. Sometimes it makes it easier to have the reminders, because you can't remember everything. The defaulters are about five to six, the reminders come on Monday morning. I can trace them by the end of day. (CHN, community health post)*

*It helps trace our important clients and the message it gave, it tells them … it saves mothers and pregnant women. (CHN, health center)*

#### 3.4.3. Broader Appeal of Aiding Policy and Managerial Decisions

Administrators and data management staff working in the district health directorates were most satisfied with the Client Data App for its capacity to summarize data for timely decision-making. The automated monthly reports provided by MOTECH enabled a new level of scrutiny for data collection and aggregation in health centers and community health posts.

*It's very good …, For instance, the fact that the data, you can get everything at the facility level. But before that came into being, everything was allocated at the health center level or sub-district level. So we're not getting the details. It is all about the details. Now we can get more detailed data at the CHPS [health post] level than used to be. I think to me that is what I can put my finger on—more details. (District health director)*

District health directors felt the Client Data App brought about a better awareness of how data were collected and used and, therefore, a better appreciation for accuracy and completeness. District health directors also noted that, for staff with limited data analytical skills, the Client Data App assisted in synthesizing data rapidly and in a user-friendly format. Having more valid, aggregated data additionally enabled administrators to monitor trends and determine what needed to be improved.

*To a large extent, because it is automated, I think I have to concede that the capacity of a good number of our staff at especially the lower levels are inadequate when you are talking about data analysis. So when it is ready-cooked like this, of course it's easy for them to—if it's [a] graphical presentation … you can visualize everything that you want. And that is very handy. (District health director)*

*As you know, these things are needed to help with decision-making … at that level. You can clearly see the trends. You can clearly see where you are doing well [and] where you are not doing well. It is so good. (District health director)*

## 4. Discussion

Our data indicate that MOTECH's Client Data App was a feasible, usable, and acceptable tool to aid health workers in collecting and tracking data to improve maternal, neonatal, and child health services. Health workers agreed that the Client Data App simplified individual client data collection, was easily integrated into their workflow, and enhanced their capacity to deliver follow-up services across the care continuum. These findings align with other studies that have demonstrated the benefits of equipping health workers with data management technologies to improve the continuity of care in rural populations [27, 61]. At the same time, our results highlight several challenges that would need to be addressed to optimize the utility of a client data management system using mobile devices in resource-poor settings.

First, our findings suggest that the successful integration of mobile applications into service delivery may necessitate targeted changes in human resources available at certain health facilities [62]. The greatest obstacle to implementation of the Client Data App was the combined effect of high client volumes, staff shortages, and poor network connectivity. CHNs tended to integrate mobile data entry into client care by waiting until the end of the day or week to upload data to the MOTECH server. Yet, the number of clients seen by health workers at the health centers and the long delays in uploading data precluded real-time submission. Thus, while data uploads were manageable for CHNs working at the community health posts, they were generally not manageable at health centers. Similar issues emerged within the context of a telemedicine program in Ghana's Amansi-West district, in which providers assumed a greater workload without a reduction in other tasks or increased personnel [63]. Our participants suggested training less skilled data staff or community health volunteers to assist with data entry, particularly for older providers who were sometimes less proficient with mobile apps [64]. In the context of future mHealth deployments, it will be important for implementing partners to work closely with the national health system and other government agencies to assess health workers' capacity to absorb additional mobile-based responsibilities and to explore possibilities for hiring and maintaining data staff.

Second, given the current cellular network in rural Ghana, it may have been possible to minimize network challenges through a more advanced monitoring system. Our results revealed that the lack of network reliability compromised the efficiency and usability of the Client Data App, notwithstanding the technological systems put in place to address these complications. Other studies have shown that poor network coverage and signal strength are often a major limitation to implementation of mobile technologies [23, 27, 31, 65, 66]. While the MOTECH team took careful measures to mitigate connectivity challenges, such as instituting a customer support call center and using high-storage devices with offline and multinetwork functionality, users still grew frustrated with the efforts required to upload data. The longs lags between data collection and submission, which CHNs reported as sometimes taking up to two weeks, limited the capacity of MOTECH to reliably track all client needs. A mechanism that allowed health workers to directly access clinical data to fix errors from incomplete uploads, rather than relying on a customer support center, may have enabled more immediate troubleshooting [40]. In addition, it is critical for implementers to carefully select what minimal clinical data are required for decision-making, rather than digitizing all paper registers or entry fields in order to maximize entry quality and speed.

Third, our findings shed light on the interconnectedness of the evaluation's assessment areas. Acceptance of the Client Data App was closely linked to its perceived usefulness to generate automated reports. When no problems were encountered during uploading, CHNs felt that the automated reports were a time saver. The MOTECH team had anticipated this would be the primary incentive for CHNs to engage with the Client Data App [46]. However, several health center-based CHNs were less motivated to enter data when the reports were not generated. These findings align with other research studies that have found technical issues such as screen freezes and delayed uploads impede efficient use and limit user uptake of mobile health applications [27, 55, 67]. A lower threshold of data completeness and accuracy for receiving automated reports may have improved usability. Similarly, engaging dedicated data staff to manage the Client Data App may have enhanced its usefulness and acceptance by increasing the number of facilities with minimum completeness standards and automated reports. Other strategies such as rewards and recognition may also have encouraged user uptake in the short term, yet their sustainability is questionable.

Studies conducted with frontline health workers prior to the integration of new technologies into their workflow have revealed high levels of acceptance and willingness to learn, despite the lack of experience with such tools [64, 68, 69]. In many cases, such interest is related to the appeal of innovative technologies as representative of modern medicine [64, 67]. Thus, efforts should be taken to capitalize on and preserve existing positive attitudes during the implementation of mobile health applications by ensuring the usability of new devices. Specifically, incurring higher upfront costs for more advanced mobile handsets may improve data accuracy and user adoption as compared to inexpensive, less user-friendly devices. MOTECH selected the Nokia 1680 model, in part, due to its lower cost (40 USD) [46] for use in Awutu Senya. Yet, as our participants concluded, the small-sized keypad was more problematic than anticipated. In later deployments of MOTECH in Gomoa West as well as the other regions not explored in this evaluation, the phone was upgraded to a more expensive, user-friendly Nokia Asha 200 [40]. The evolution of mobile phones used by MOTECH highlights the value of continually assessing user experiences and reevaluating the trade-offs inherent in each decision. As the participants noted, using larger tablets or laptops in the future may further improve client data management at facility and community levels.

Finally, greater global focus has been placed on ensuring the interoperability of mobile and digital health innovations [38]. The proliferation of multiple disconnected mHealth systems in many countries has led to calls to examine how systems operating in the same country can exchange information across multiple platforms. Systems like OpenHIE (https://ohie.org/) and similar “shared health record” alliances are being explored as a mechanism to allow smaller mHealth products to share connectivity to a central backbone of core information. In our evaluation, participants identified a lack of interoperability with the national DHIMS-2 as an important barrier to maximizing utility. Although often more expensive and time-consuming during development, the future benefits of building mobile applications to global health information standards quickly materialize with increased scale, through interoperability with other facility and national aggregate systems.

*Limitations and Strengths*. This evaluation was limited by the following factors. First, self-reported data is subject to social desirability bias, and thus participants may have exaggerated their positive reactions to the Client Data App. However, this bias may have been minimized by the fact that the interviewers were external consultants and not affiliated with MOTECH. Second, the transferability of the findings to other MOTECH districts may be limited by the unique situation of the Awutu Senya site. For example, the district health director from Awutu Senya was highly dedicated to and involved in project implementation, which may not be true in other settings. Nevertheless, by engaging with diverse user groups, this evaluation provided rich insights into the Client Data App's implementation challenges from multiple perspectives. The use of two types of qualitative methods further strengthened the credibility of the findings, as later-stage interviews and the focus groups were able to provide feedback on early analyses.

## 5. Conclusion

Mobile phones hold great promise for overcoming health disparities among rural populations by bridging the gap between access to client health information and service provision. MOTECH's Client Data App is a promising tool to aid health workers in collecting and tracking data across the health care continuum. Results of this evaluation may be used to guide future research on mHealth innovations to address challenges related to infrastructure, human resources, and technology before and during program deployment. Qualitative assessments of user perceptions should remain a priority in efforts to optimize the use of mobile data applications to alleviate barriers to maternal, neonatal, and child care in Ghana and beyond.

## Figures and Tables

**Table 1:** Number and type of users enrolled in the evaluation by qualitative method and by total.

User characteristics	Individual interviews	Focus groups	Total
Total number of interviews or discussions	14	2	16

Number of interviews or discussions by health system level			
Health center	4	0	4
Community health post	6	2	8
District health directorate	4	0	4

Total number of enrolled users	14	15	29

Number of enrolled users by health worker type			
Community health nurse	8	15	23
Midwife	2	0	2
District health director	2	0	2
District health information officer	2	0	2
Number of enrolled users by age (in years)			
<26	0	2	2
26–29	7	13	20
≥30	7	0	7
Number of enrolled users by gender			
Female	11	13	24
Male	3	2	5
